# A High-Fat/High-Protein, Atkins-Type Diet Exacerbates *Clostridioides* (*Clostridium*) *difficile* Infection in Mice, whereas a High-Carbohydrate Diet Protects

**DOI:** 10.1128/mSystems.00765-19

**Published:** 2020-02-11

**Authors:** Chrisabelle C. Mefferd, Shrikant S. Bhute, Jacqueline R. Phan, Jacob V. Villarama, Dung M. Do, Stephanie Alarcia, Ernesto Abel-Santos, Brian P. Hedlund

**Affiliations:** a School of Life Sciences, University of Nevada, Las Vegas, Las Vegas, Nevada, USA; b Department of Chemistry and Biochemistry, University of Nevada, Las Vegas, Las Vegas, Nevada, USA; c Nevada Institute of Personalized Medicine, University of Nevada, Las Vegas, Las Vegas, Nevada, USA; MIT

**Keywords:** Atkins diet, *Clostridium difficile*, microbiome

## Abstract

Clostridioides difficile (formerly Clostridium difficile) infection (CDI) can result from the disruption of the resident gut microbiota. Western diets and popular weight-loss diets drive large changes in the gut microbiome; however, the literature is conflicted with regard to the effect of diet on CDI. Using the hypervirulent strain C. difficile R20291 (RT027) in a mouse model of antibiotic-induced CDI, we assessed disease outcome and microbial community dynamics in mice fed two high-fat diets in comparison with a high-carbohydrate diet and a standard rodent diet. The two high-fat diets exacerbated CDI, with a high-fat/high-protein, Atkins-like diet leading to severe CDI and 100% mortality and a high-fat/low-protein, medium-chain-triglyceride (MCT)-like diet inducing highly variable CDI outcomes. In contrast, mice fed a high-carbohydrate diet were protected from CDI, despite the high levels of refined carbohydrate and low levels of fiber in the diet. A total of 28 members of the *Lachnospiraceae* and *Ruminococcaceae* decreased in abundance due to diet and/or antibiotic treatment; these organisms may compete with C. difficile for amino acids and protect healthy animals from CDI in the absence of antibiotics. Together, these data suggest that antibiotic treatment might lead to loss of C. difficile competitors and create a favorable environment for C. difficile proliferation and virulence with effects that are intensified by high-fat/high-protein diets; in contrast, high-carbohydrate diets might be protective regardless of the source of carbohydrate or of antibiotic-driven loss of C. difficile competitors.

**IMPORTANCE** The role of Western and weight-loss diets with extreme macronutrient composition in the risk and progression of CDI is poorly understood. In a longitudinal study, we showed that a high-fat/high-protein, Atkins-type diet greatly exacerbated antibiotic-induced CDI, whereas a high-carbohydrate diet protected, despite the high monosaccharide and starch content. Our study results, therefore, suggest that popular high-fat/high-protein weight-loss diets may enhance CDI risk during antibiotic treatment, possibly due to the synergistic effects of a loss of the microorganisms that normally inhibit C. difficile overgrowth and an abundance of amino acids that promote C. difficile overgrowth. In contrast, a high-carbohydrate diet might be protective, despite reports on the recent evolution of enhanced carbohydrate metabolism in C. difficile.

## Abstract (precis)

The role of Western and weight-loss diets with extreme macronutrient composition in the risk and progression of CDI is poorly understood. In a longitudinal study, we showed that a high-fat/high-protein, Atkins-type diet greatly exacerbated antibiotic-induced CDI, whereas a high-carbohydrate diet protected, despite the high monosaccharide and starch content. Our study results, therefore, suggest that popular high-fat/high-protein weight-loss diets may enhance CDI risk during antibiotic treatment, possibly due to the synergistic effects of a loss of the microorganisms that normally inhibit C. difficile overgrowth and an abundance of amino acids that promote C. difficile overgrowth. In contrast, a high-carbohydrate diet might be protective, despite reports on the recent evolution of enhanced carbohydrate metabolism in C. difficile.

## INTRODUCTION

Clostridioides difficile (formerly Clostridium difficile) is an endospore-forming member of the phylum *Firmicutes* that is the leading cause of antibiotic-associated and hospital-acquired diarrhea. C. difficile infections (CDIs) make up >70% of health care-associated gastrointestinal infections, with symptoms ranging from mild diarrhea in mild infections to ulcerative colitis and toxic megacolon in severe infections (1). Moreover, CDI is financially taxing on U.S. hospital management (2) and is the cause of over 500,000 diagnosed cases and 29,000 deaths annually, according to a 2015 report (3).

Stable and complex microbial communities in the gut act as a natural barrier against C. difficile (4), but broad-spectrum antibiotics can disrupt the native microflora, allowing C. difficile to multiply and cause CDI (5). Importantly, C. difficile has innate resistance to multiple antibiotics and CDI is closely linked to administration of ampicillin, amoxicillin, cephalosporins, clindamycin, and fluoroquinolones (6). In order to cause successful infection, C. difficile spores must germinate, grow within the intestinal lumen, and produce toxins that mediate tissue damage and inflammation (7). Specific chemical signals are needed for each of these steps. For example, spore germination is promoted by variety of amino acids and primary bile salts but is inhibited by secondary bile salts (8); growth can be supported by fermentation of amino acids or carbohydrates but is inhibited by short-chain fatty acid (SCFA) products of carbohydrate fermentation (9–11); finally, toxin production is inhibited by several amino acids, particularly cysteine (12). Thus, it is logical that diet might affect the incidence and severity of CDI, and yet the literature is contradictory on relationships between diet and CDI.

Several studies have suggested that high-carbohydrate/low-protein diets can mitigate antibiotic-induced CDI. Moore et al. (13) hypothesized that poor nutrient status would worsen CDI but instead found that protein-deficient diets (with increased carbohydrate content) mitigated CDI severity in C57BL/6 mice infected with hypervirulent strain VPI 10463 (ribotype 078 [RT078]). Another study using humanized mice inoculated from antibiotic-induced dysbiotic subjects reported increased lumen amino acid concentrations and severe CDI compared with those inoculated from control subjects (11). The same study showed that C. difficile strain 630 expressed the proline reductase, PrdA, only in dysbiotic mice and that *prdB* mutants unable to use proline as the Stickland electron acceptor failed to colonize mice. Finally, they showed that low-protein and, specifically, low-proline diets lessened colonization and virulence. A separate study found that mixtures of microbiota-accessible carbohydrates (MACs), or, specifically, inulin, decreased C. difficile burdens in humanized mice, while stimulating growth of carbohydrate-utilizing microbes and SCFA production (10).

In contrast, other studies have implicated carbohydrates, specifically, simple sugars, in the proliferation of hypervirulent, epidemic C. difficile strains. One study reported on the independent evolution of mechanisms to utilize the artificial sweetener trehalose in RT027 and RT078 and showed that trehalose supported growth of these ribotypes *in vitro* (14). Trehalose also increased toxin production and decreased survival in a humanized mouse model of CDI but did not increase C. difficile burden. Similarly, Kumar and colleagues (15) reported positive evolutionary selection in the fructose phosphotransferase system and for several other enzymes involved in the transport and fermentation of simple sugars in recently evolved strains of C. difficile, including RT027. That study also reported that glucose and fructose enhanced growth and sporulation of RT027 *in vitro* and shedding in a mouse model of CDI, but relationships between dietary monosaccharides and virulence were not reported.

Western diets would seem to favor CDI since they are enriched in both proteins and simple sugars and yet are deficient in MACs and other fiber sources (16), and rates of CDIs are indeed highest in developed countries (17). Modern weight-loss diets such as Atkins and ketogenic diets are extreme because the majority of calories are from fat and protein and because carbohydrates typically contribute less than 10% of caloric intake (18, 19). These diets have been wildly popular; for example, Dr. Atkins’ “Diet Revolution” is the best-selling diet book in history (20). Keto diets are similar to Atkins’ diets but tend to be more extreme, reducing both dietary carbohydrate and protein levels. And yet, despite the increasing evidence tying C. difficile evolution and pathogenesis to diet and the continuing revolution of modern diets with extreme macronutrient composition, diet has not been featured as a major factor in models of CDI (17).

Here, we assessed the effect of diet, including a high-fat/high-protein Atkins-like diet, a high-fat/low-protein keto-like diet resembling the medium-chain-triglyceride (MCT) diet, and a high-carbohydrate diet, on the outcome of antibiotic-associated CDI using hypervirulent C. difficile strain R20291 and described concomitant changes in microbial community diversity and composition.

## RESULTS

### A high-fat/high-protein diet exacerbates CDI, and yet a high-carbohydrate diet provides protection.

**FIG 1:**
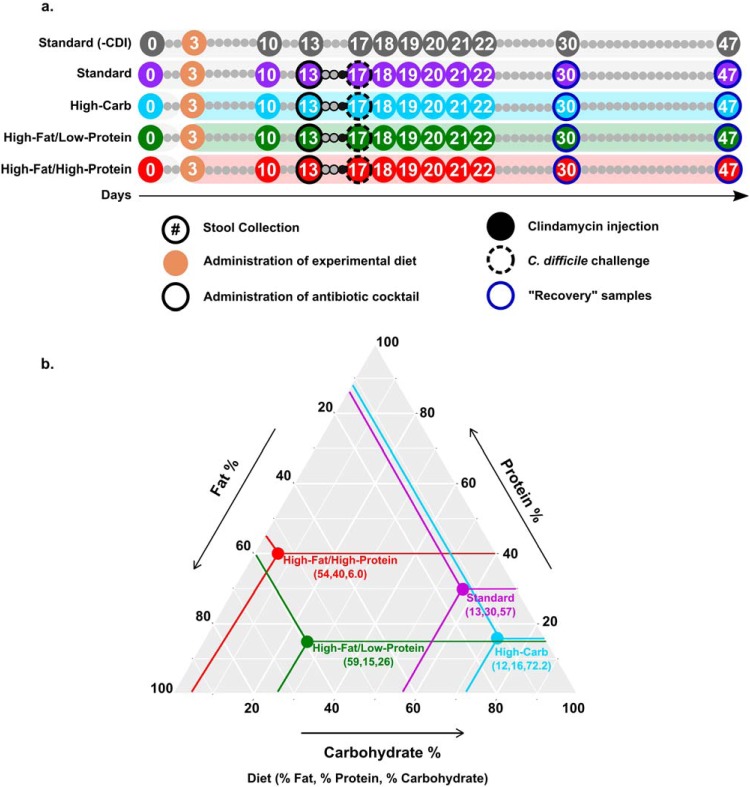
Experimental timeline and macronutrient contents of the diets. (a) High-carbohydrate (blue), high-fat/low-protein (green), or high-fat/high-protein (red) diets were introduced on day 3. An antibiotic cocktail (solid outline) and clindamycin (black-filled circles) were given on day 13 and day 16, respectively. Mice were challenged with C. difficile R2027 spores on day 17 (dashed outline). Circles with numbers indicate the days on which fecal samples were collected. Stool collection took place prior to manipulation of mice or experimental treatment. (b) A ternary plot depicting micronutrient contents (% Fat, % Protein, % Carbohydrate) of high-carbohydrate (blue), high-fat/low-protein (green), high-fat/high-protein (red), and standard laboratory (purple) diets.

**FIG 2:**
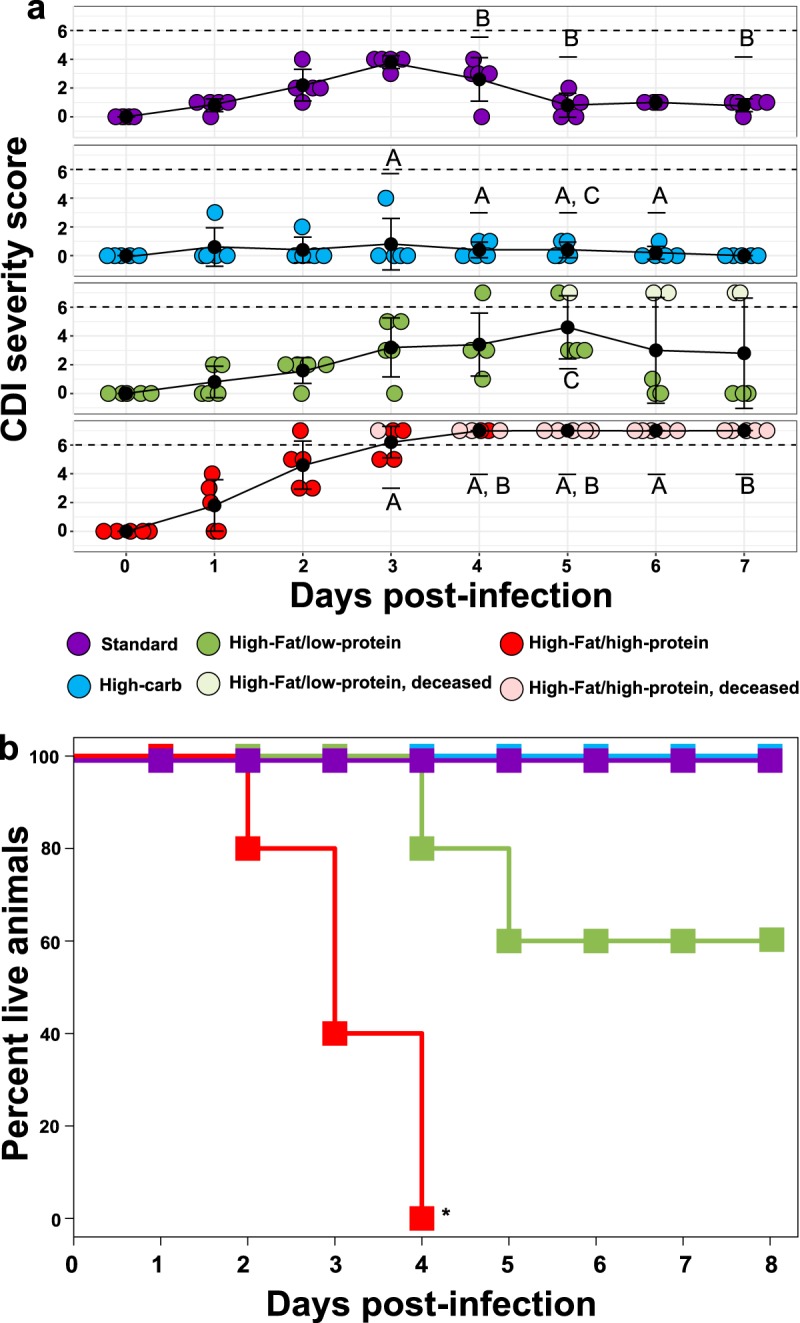
Effect of diet on mouse survival and CDI severity postinfection. (a) Mean disease severity scores (black dots with connected black trendline) for each diet following CDI challenge; 25th and 75th percentiles are shown. Colored dots represent severity scores for individual mice. Dashed lines represent a score of 6, the clinical endpoint. Groups marked A, B, or C indicate statistically significant differences (*P < *0.05, two-way repeated-measures [ANOVA]) in disease severity between mice fed a high-carbohydrate diet versus a high-fat/high-protein diet (letter A), a standard laboratory diet versus a high-fat/high-protein diet (letter B), or a high-carbohydrate diet versus high-fat/low-protein diet (letter C). There were significant (*P ≤ *0.001, two-way repeated-measures ANOVA, **) changes in CDI severity through time in infected mice fed a standard laboratory diet and a high-fat/high-protein diet. (b) Kaplan-Meier survival curves for mice fed a high-carbohydrate diet (blue, *n* = 5), high-fat/low-protein diet (green, *n* = 5), high-fat/high-protein diet (red, *n* = 5), and standard laboratory diet (purple, *n* = 5), all following CDI challenge. The high-fat/high-protein diet significantly (*P = *0.003, log rank test, *) reduced survival of infected mice. All uninfected mice fed the standard laboratory diet showed no CDI signs (score of 0) for the duration of the experiment (not shown).

To determine whether diet affects the progression of CDI, groups of five mice were fed diets differing in macronutrient composition (Fig. 1) as follows: a high-fat/high-protein diet, a high-fat/low-protein diet, a high-carbohydrate diet, and a standard laboratory diet (see Table S1 to S4 in the supplemental material). To quantify the effect of the diets on CDI severity, morbidity and mortality were examined over the course of the experiment (Fig. 2) using established metrics (21) with amendments as described in Materials and Methods. All infected mice fed the standard laboratory diet developed mild CDI signs but eventually recovered. The mean time of CDI sign onset was 2.8 ± 0.4 days, and the mean recovery time was 4.8 ± 0.4 days. In contrast, only two of the mice fed the high-carbohydrate diet exhibited mild symptoms, and they quickly recovered. The mean time of CDI sign onset was 2.0 ± 1.8 days, and the mean recovery time was 3.0 ± 1.3 days. The rest of the animals in this group never developed any CDI signs. Mice fed the high-fat/low-protein diet showed CDI symptom onset heterogeneity. Two animals developed severe CDI and became moribund. Meanwhile, three animals developed mild to moderate CDI signs similar to those seen with the standard diet and recovered within a week postinfection. The mean time of CDI sign onset was 3.2 ± 0.4 days, and the mean recovery time was 6.0 ± 0.0 days. Strikingly, all mice fed the high-fat/high-protein diet developed severe CDI signs and were euthanized within 4 days following C. difficile challenge. For these mice, the mean time of CDI sign onset was 1.6 ± 0.5 days. The difference in survival rates between the mice fed the high-fat/high-protein diet and all other animal groups was significant (*P = *0.003 [log rank test]). Surviving animals in all groups resolved all CDI signs (score of 0) within 8 days and remained healthy for the remainder of the experiment. A control cohort of uninfected mice fed the standard laboratory diet remained healthy and showed no CDI signs for the duration of the experiment (not shown).

10.1128/mSystems.00765-19.7 TABLE S1 Comparison of macronutrient compositions of tested diets. Download Table S1, PDF file, 0.1 MB. Copyright © 2020 Mefferd et al. 2020 Mefferd et al. This content is distributed under the terms of the Creative Commons Attribution 4.0 International license.

### Reduced microbial diversity is associated with changes in diet, antibiotic treatment, and CDI.

**FIG 3:**
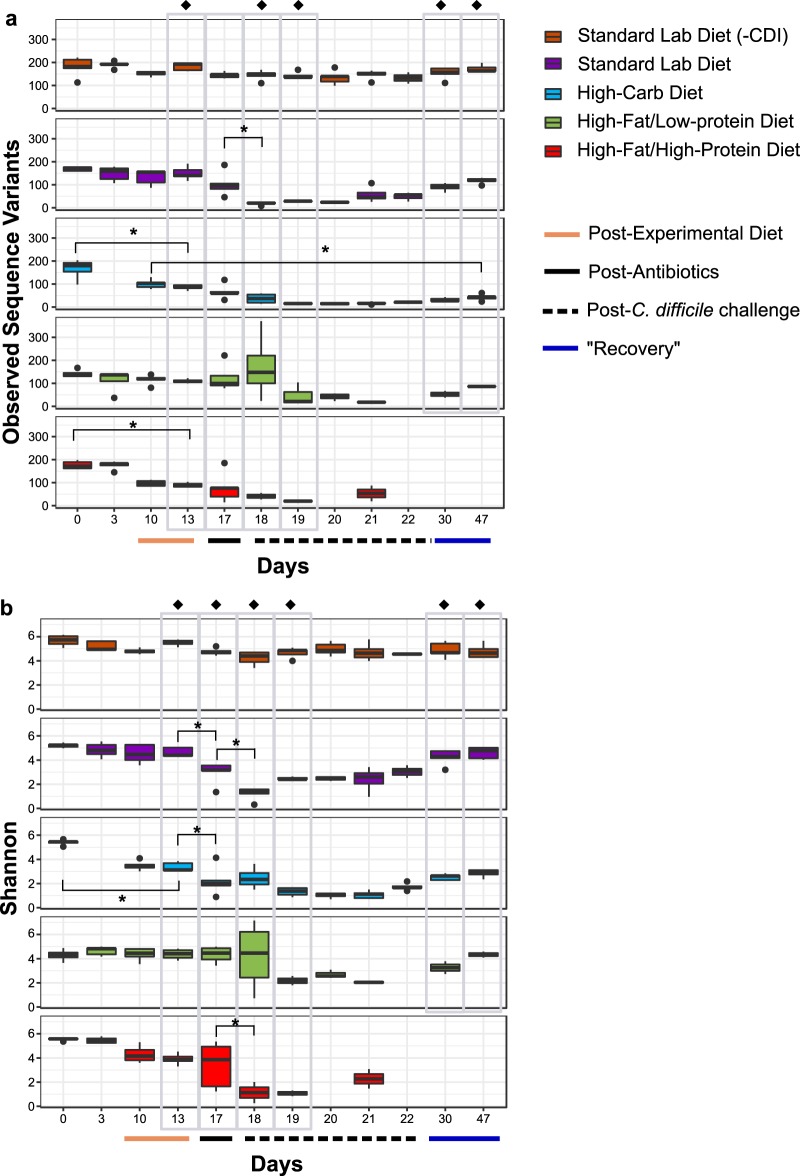
Effect of diet and treatment on alpha diversity. (a) Observed sequence variants (SV) and (b) Shannon diversity were calculated for uninfected mice fed a standard laboratory diet (orange, *n* = 5) and for infected mice fed a standard laboratory diet (purple, *n* = 5), high-carbohydrate diet (blue, *n* = 5), high-fat/low-protein diet (green, *n* = 5), or high-fat/high-protein diet (red, *n* = 5). Gray boxes highlight comparisons between groups after a change in diet on day 13 and antibiotic treatments on day 17, postinfection on days 18 and 19, and recovery on days 30 and 47. Administration of experimental diets (solid tan line, *x* axis) and time points after antibiotics administration (solid black line, *x* axis) and C. difficile challenge (dashed black line, *x* axis) are indicated. Black dots above and below box plots represent outliers. Asterisks (*) indicate significant (*P < *0.05) loss of diversity in within-group pairwise comparisons. Filled diamonds (◆) indicate significant (*P < *0.05 [ANOVA]) differences between groups on a given day.

To understand changes in gut microbial diversity due to experimental manipulations, alpha diversity was analyzed, as measured by richness (observed sequence variants [SVs]) (Fig. 3a), Simpson’s evenness (see Fig. S1 in the supplemental material), and Shannon diversity (Fig. 3b) over the course of the experimental timeline. All animal groups showed significant (*P < *0.05 [analysis of variance {ANOVA}]) change in diversity over time, due to decreases in richness and evenness corresponding to changes in diet (day 13) or antibiotic treatment (day 17) and/or disease status (days 18 and 19).

10.1128/mSystems.00765-19.1 FIG S1 Effect of diet and treatment on alpha diversity—Simpson’s evenness. Simpson’s evenness was calculated for uninfected mice fed a standard laboratory diet (orange, *n* = 5) and for infected mice fed a standard laboratory diet (purple, *n* = 5), a high-carbohydrate diet (blue, *n* = 5), a high-fat/low-protein diet (green, *n* = 5), or a high-fat/high-protein diet (red, *n* = 5). Gray boxes highlight comparisons between groups after a change in diet on day 13, antibiotic treatments on day 17, postinfection on days 18 and 19, and recovery on days 30 and 47. Administration of experimental diets (solid tan line, *x* axis) and time points after antibiotics administration (solid black line, *x* axis) and C. difficile challenge (dashed black line, *x* axis) are indicated. Asterisks (*) indicate significant (*P < *0.05) loss of diversity in within-group pairwise comparisons. Filled diamonds (◆) indicate significant (*P < *0.05 [ANOVA]) differences between groups on a given day. Download FIG S1, EPS file, 0.2 MB. Copyright © 2020 Mefferd et al. 2020 Mefferd et al. This content is distributed under the terms of the Creative Commons Attribution 4.0 International license.

Specifically, there were significant differences (*P < *0.05 [ANOVA]) in richness, evenness, and Shannon diversity between the diet groups after mice were fed the experimental diets for 10 days (day 13), exemplified by significant decreases in richness and Shannon diversity after administration of the high-carbohydrate diet (day 0 versus day 13) (*P < *0.05 [ANOVA and Tukey’s honestly significant difference {HSD} test]) and richness after administration of the high-fat/high-protein diet (day 0 versus day 13) (*P < *0.05 [ANOVA and Tukey’s HSD test]). Diversity results were also distinct in the diet groups following antibiotic treatment; in particular, there was a significant loss of diversity after antibiotic treatments (day 13 versus day 17) (*P < *0.05 [ANOVA and Tukey’s HSD test]) in mice fed the standard laboratory diet (evenness and Shannon diversity) and the high-carbohydrate diet (Shannon diversity). The diet groups were also distinct with regard to all three diversity indices following inoculation with spores (day 18 and day 19), and there were significant losses in diversity in mice fed the standard laboratory diet and the high-fat/high-protein diet corresponding to CDI development (day 17 versus day 18) (*P < *0.05 [ANOVA and Tukey’s HSD test]).

For mice fed the standard and high-fat/low-protein diets, most alpha diversity metrics returned to their diet-acclimated states within 30 days post-C. difficile challenge (day 13 versus day 47) (*P > *0.05). In contrast, gut microbiome richness did not return to normal in mice fed the high-carbohydrate diet over this time course (day 10 versus day 47) (*P < *0.05).

### Microbial communities transitioned through a common pattern in response to experimental manipulations.

**FIG 4:**
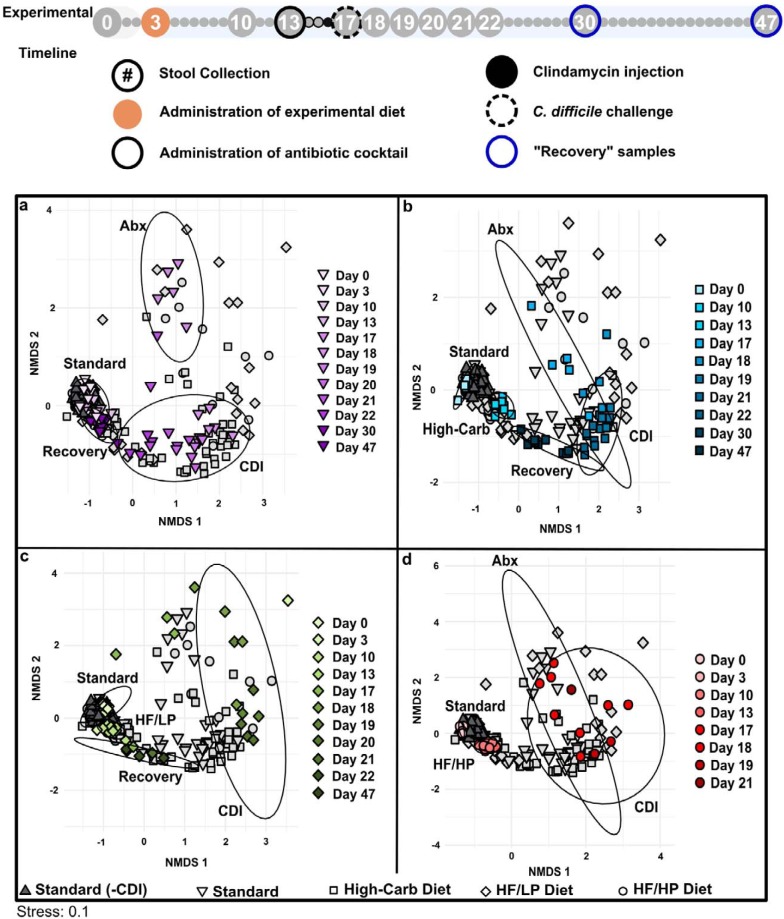
NMDS analysis based on Bray-Curtis dissimilarity. Each panel presents a visualization of the same data and highlights the analysis for infected mice fed a (a) standard laboratory diet, (b) high-carbohydrate diet (blue), (c) high-fat/low-protein diet (HF/LP, green), and (d) high-fat/high-protein diet (HF/HP, red). Colors are shaded to show time progression through the experiment. Data representing uninfected mice fed a standard laboratory diet (dark gray triangles) are featured in all panels. Ellipses represent standard errors of the mean (95% confidence) for samples associated with the standard laboratory diet (labeled “Standard,” days 0 and 3), diet-associated microbiomes (days 10 and 13), antibiotic treatments (labeled “Abx,” day 17), CDI (days 18 to 22), and recovery (days 30 and 47) for the colored data points associated with each experimental group. A 95% confidence ellipse of samples representing mice fed a high-fat/low-protein diet (panel c) on day 17 was not included, as it was large and included nearly all points in the data set. This indicates that there was variability in the high-fat/low-protein samples after antibiotic treatments, and results must be interpreted with caution due to small sample size. For guidance, an amended experimental timeline image from Fig. 1 is included.

To assess microbial community changes over the experimental timeline, Bray-Curtis dissimilarity was calculated and visualized by nonmetric multidimensional scaling (NMDS). This analysis revealed a common pattern of microbial community transition through the experimental time course in all groups for the following parameters: diet-associated state, antibiotic-associated state (Abx), CDI-associated state (CDI), and recovery state (recovery) (Fig. 4). Although overall patterns in the progression of these states were similar, some diet-specific effects were apparent.

Infected mice fed the standard laboratory diet progressed through distinct phases of transition and returned to a quasi-pre-CDI community structure, as indicated by overlapping the “Standard” and “Recovery” confidence ellipses (Fig. 4a). However, some microbial groups did not return after the experimental treatments, exemplified by the phylum *Tenericutes* (Fig. S2 to S5). In contrast, the diet-associated and recovery ellipses did not overlap in mice fed the high-carbohydrate and high-fat/low-protein diets (Fig. 4b and c), indicating incomplete restoration of the gut microbial communities. The analysis also highlighted the large variability in microbial community structure in mice fed the high-fat/low-protein diet following antibiotic treatment (Fig. 4c), which was generally consistent with the heterogeneous CDI outcomes of the mice on that diet. A recovery phase was not observable in mice fed the high-fat/high-protein diet due to 100% mortality of these mice by day 21 (Fig. 4d). Uninfected mice fed the standard laboratory diet clustered together throughout the experiment.

10.1128/mSystems.00765-19.2 FIG S2 Bar plot of relative abundances—standard laboratory diet. The bar plot shows relative abundances of gut microbiota at the phylum level in each mouse fed the standard laboratory diet across the experimental timeline. Samples associated with the days that mice were fed a standard laboratory diet and administered antibiotics and with time points postinfection and during recovery are indicated. Download FIG S2, EPS file, 0.5 MB. Copyright © 2020 Mefferd et al. 2020 Mefferd et al. This content is distributed under the terms of the Creative Commons Attribution 4.0 International license.

### Diet impacted antibiotic- and recovery-associated microbial communities.

**FIG 5:**
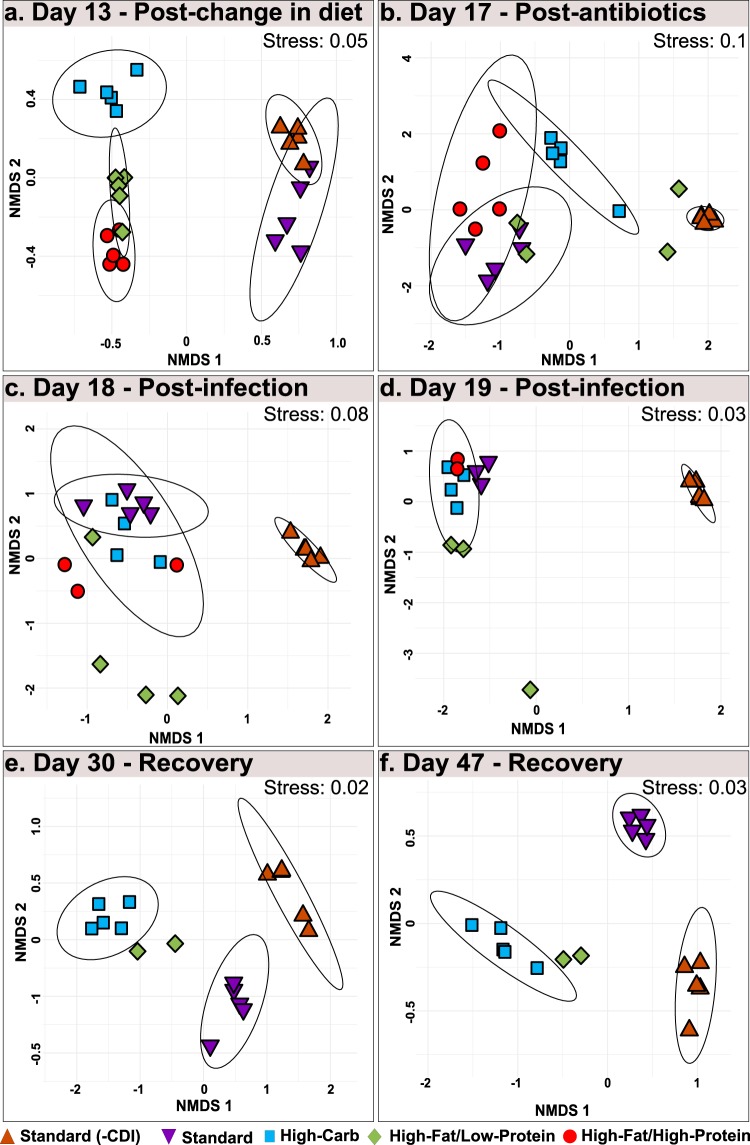
NMDS analysis based on Bray-Curtis dissimilarity for days 13, 17, 18, 19, 30, and 47. Each panel presents an ordination of samples from uninfected mice fed a standard laboratory diet (orange) and from infected mice fed a standard laboratory diet (purple), high-carbohydrate diet (blue), high-fat/low-protein diet (green), or high-fat/high-protein diet (red) for days (a) 13, (b) 17, (c) 18, (d) 19, (e) 30, and (f) 47. Ellipses represent standard errors of the mean (95% confidence). Data representing 95% confidence are not shown for the mice fed a standard laboratory diet and high-fat/low-protein diet on days 17, 18, and 19, as the associated data fields were large and included nearly all points in the data set. Also, 95% confidence ellipses are not shown for mouse groups with significant mortality, as the calculation cannot compute with *n* = <4.

To better investigate the effect of diet on microbial community response to specific treatments, Bray-Curtis dissimilarity was calculated for crucial time points in the experiment (Fig. 5). Diet-specific microbial communities developed prior to antibiotic treatment (day 13), as indicated by nonoverlapping ellipses and highly significant analysis of similarity (ANOSIM) values (*P < *0.05; *R* > 0.912) for standard, high-carbohydrate, and high-fat/high-protein diets on day 13; however, the community structures of high-fat/low-protein-fed animals were not distinct (Fig. 5a).

The distinctness of the microbial communities was disrupted by antibiotic treatment and CDI, as evidenced by overlapping ellipses and insignificant ANOSIM values for day 17, day 18, and day 19, indicating the dominant role of the antibiotic treatments and CDI over the diet treatments in structuring the microbial community (Fig. 5b to d). Following recovery, diet-specific clustering patterns reemerged in the recovery phase on day 30 and day 47 (Fig. 5e and f).

Similar to CDI severity signs, the high-fat/low-protein microbiomes were heterogeneous during these treatments. However, no connection was observed between individual animal changes in microbial communities and disease severity or onset.

### Diet and antibiotics administration profoundly altered the microbiome composition.

**FIG 6:**
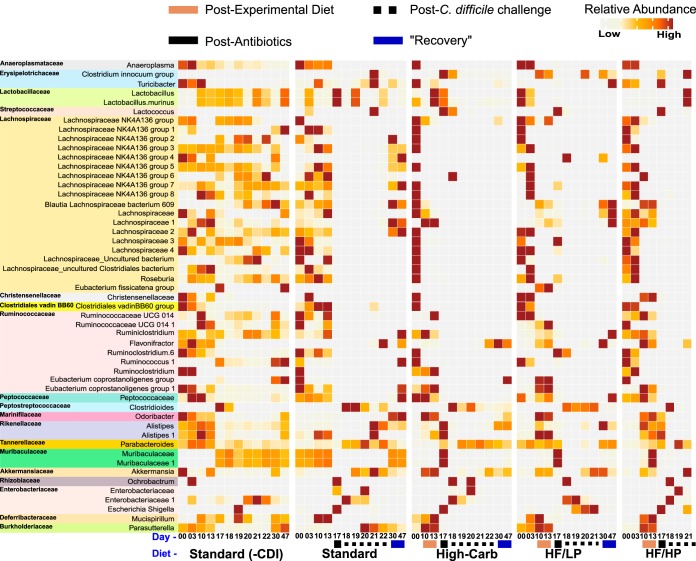
SIMPER analysis results displaying top SVs responsible for dissimilarity between experimental groups. The heat map indicates the mean relative abundances of 51 SVs that contributed cumulatively to 50% of community dissimilarities at each time point among the mice fed the standard laboratory diet, high-carbohydrate diet (High-Carb), high-fat/low-protein diet (HF/LP), and high-fat/high-protein diet (HF/HP). Each square represents the mean relative abundance of the given SV on a particular day for a particular diet. Higher intensity of brown coloring correlates with higher relative abundance.

Similarity percent (SIMPER) analysis (22) identified 51 SVs that contributed to 50% of microbial community dissimilarity between all pairwise comparisons of the diet-specific microbiomes throughout the experiment (Fig. 6). More than half of these SVs belonged to the *Clostridiales*, predominantly the families *Lachnospiraceae* (19/51) and *Ruminococcaceae* (9/51), and were dominated by uncultivated genera. Most *Lachnospiraceae* SVs decreased in abundance after administration of the experimental diets, particularly the high-fat/low-protein diet and the high-carbohydrate diet, and were further reduced following antibiotic treatment and CDI. The *Ruminococcaceae* SVs were more variable in response to the diets but were also strongly depleted following antibiotic treatment.

Several other groups also showed strong patterns. We compared the relative abundances of these taxa at key time points using ANOVA and Tukey’s HSD test and found significant differences in their abundances with respect to uninfected mice across the experimental timeline. Two *Muribaculaceae* SVs became depleted in abundance in the high-carbohydrate and high-fat/low-protein groups but then bloomed (day 17) (*P* < 0.05) and crashed following antibiotic treatment and infection (days 18 and 19) (*P* < 0.05) and never recovered (days 30 and 47) (*P* < 0.05). Also, two *Alistipes* SVs slightly increased in abundance after administration of the experimental diets (day 17) (*P* < 0.05), followed by a reduction after antibiotic treatment and/or CDI. An SV affiliated with the Clostridium innocuum group emerged at different times after the antibiotic treatment in all the antibiotic-treated mice. Further, abundances of some members of the *Proteobacteria*, including *Escherichia*/*Shigella* and an uncultivated member of the *Enterobacteriaceae*, expanded after the antibiotic treatment, and yet abundances of *Parasutterella* decreased in all antibiotic-treated mice (days 17 and 18) (*P* < 0.05). Also, an SV in the order *Parabacteroides* expanded after the antibiotic treatment (day 19) (*P* < 0.05) in all but the high-fat/low-protein diet treatments. The relative abundance of *Akkermansia* increased after C. difficile challenge (day 18 and 21) (*P* < 0.05) irrespective of diet.

## DISCUSSION

The mammalian gut microbiota is crucial for host health and provides colonization resistance against various enteric pathogens (23). Exposure to broad-spectrum antibiotics leads to the depletion of commensal microbiota, an effect which can be exploited by pathogens such as C. difficile (24). Diet is an important force that determines gut microbial composition and function (25). Consequently, several studies have demonstrated the effects of dietary components on C. difficile growth, physiology, and pathogenesis both *in vitro* and in antibiotic-induced animal models of CDI; however, those studies have been contradictory regarding the relative importance of proteins and carbohydrates in effects on CDI. The goal of this study was to broadly assess CDI outcomes and microbial community responses to diets with extreme differences in macronutrient composition following antibiotic treatment.

### Effect of high-carbohydrate diets on antibiotic-induced CDI.

Improved gut health due to high-carbohydrate diets, especially those rich in fiber, has been well documented and is suggested to be related to the production of SCFAs by gut microbes (26–30). Some studies have pointed to the importance of MACs (specifically, inulin) in mitigation of CDI (10); however, the inulin content of the high-carbohydrate diet in our study was low (2.1% [wt/vol]). Instead, the major sources of carbohydrates were corn starch (43.5% [wt/vol]), maltodextrin (14.4% [wt/vol]), and sucrose (11.0% [wt/vol]). The highly digestible corn starch and maltodextrin incorporated in these diets would be depolymerized to monosaccharides readily. Thus, our study results suggest that a high-carbohydrate diet that is correspondingly low in protein can be protective against CDI, irrespective of the specific carbohydrate composition.

This result superficially contradicts reports of recent adaptations enhancing the transport, metabolism, and general physiology of different strains of C. difficile in response to glucose, fructose, and trehalose (14, 15). However, the study on glucose and fructose metabolism did not describe any effects of sugars on the pathogenesis of RT027. Instead, they reported that monosaccharides promoted growth and sporulation *in vitro* and colonization in a mouse model of CDI. Thus, it appears that monosaccharides or easily digestible polysaccharides might promote C. difficile colonization while simultaneously limiting CDI overgrowth and pathogenesis. Indeed, we found that mice fed the high-carbohydrate diet became long-term carriers of low-abundance populations of C. difficile, whereas mice fed the standard and high-fat/low-protein diets cleared C. difficile within 30 days post-C. difficile challenge (see Fig. S6 in the supplemental material). These studies paint a somewhat complicated picture with regard to the effects of dietary sugars on C. difficile and suggest that those effects should be addressed using carefully controlled and documented experiments that take into account the strain of C. difficile and the precise diet used in the animal model. In this regard, it is noteworthy that recent studies on the effects of sugars on C. difficile have not reported the diet used for the experiments (14, 15). In summary, the available data suggest the following: (i) any dietary sugar protects from antibiotic-associated CDI; (ii) monosaccharides and easily digestible polysaccharides might promote low-level C. difficile colonization that might be important for C. difficile epidemiology; and (iii) MACs might simultaneously protect against CDI and select against C. difficile colonization.

10.1128/mSystems.00765-19.3 FIG S3 Bar plot of relative abundances—high-carbohydrate diet. The bar plot shows relative abundances of gut microbiota at the phylum level in each mouse given the high-carbohydrate diet across the experimental timeline. Samples associated with the days that mice were fed a standard laboratory diet, switched to the high-carbohydrate diet, and administered antibiotics and with time points postinfection and during recovery are indicated. Download FIG S3, EPS file, 0.4 MB. Copyright © 2020 Mefferd et al. 2020 Mefferd et al. This content is distributed under the terms of the Creative Commons Attribution 4.0 International license.

10.1128/mSystems.00765-19.4 FIG S4 Bar plot of relative abundances—high-fat/low-protein (HF/LP) diet. The bar plot shows relative abundances of gut microbiota at the phylum level in each mouse given the high-fat/low-protein (HF/LP) diet across the experimental timeline. Samples associated with the days that mice were fed a standard laboratory diet, switched to the high-fat/low-protein diet, and administered antibiotics and with time points postinfection and during recovery are indicated. Download FIG S4, EPS file, 0.4 MB. Copyright © 2020 Mefferd et al. 2020 Mefferd et al. This content is distributed under the terms of the Creative Commons Attribution 4.0 International license.

10.1128/mSystems.00765-19.5 FIG S5 Relative abundance bar plot—high-fat/high-protein (HF/HP) diet. Bar plot showing the relative abundance of gut microbiota at the phylum level in each mouse given the high-fat/high-protein (HF/HP) diet across the experimental timeline. Samples associated with the days that mice were fed a standard laboratory diet, switched to the high-fat/high-protein diet, and administered antibiotics and with time points postinfection and during recovery are indicated. Download FIG S5, EPS file, 0.3 MB. Copyright © 2020 Mefferd et al. 2020 Mefferd et al. This content is distributed under the terms of the Creative Commons Attribution 4.0 International license.

10.1128/mSystems.00765-19.6 FIG S6 Log relative abundances of *Clostridioides* across experimental timeline. Log relative abundances of *Clostridioides* were calculated for uninfected mice fed a standard laboratory diet (orange) and for infected mice fed a standard laboratory diet (purple, *n* = 5), a high-carbohydrate diet (blue, *n* = 5), a high-fat/low-protein diet (green, *n* = 5), or a high-fat/high-protein diet (red, *n* = 5). Download FIG S6, EPS file, 0.2 MB. Copyright © 2020 Mefferd et al. 2020 Mefferd et al. This content is distributed under the terms of the Creative Commons Attribution 4.0 International license.

### Effect of a high-fat/high-protein, Atkins-like diet on antibiotic-induced CDI.

Our experiments revealed that CDI was exacerbated in mice fed high-fat diets, particularly in mice fed a high-fat/high-protein, Atkins-like diet. A high-fat diet was also observed to intensify CDI in a hamster model of infection (31); however, to our knowledge, a high-fat/high-protein diet has never been explored in animal models of CDI, as typical diets used for mouse and hamster models of CDI have ≤10% kcal from fat and ≤30% kcal from protein. The poor CDI outcome observed here for mice fed the high-fat/high-protein diet compared with the high-fat/low-protein diet is consistent with known relationships between dietary protein and CDI in both laboratory mice with native murine microbiota (13) and humanized mice fed a defined amino acid diet deficient in the Stickland electron acceptor proline (11). The major diets used in these other studies used relatively low (≤20%) concentrations of protein provided as casein. The protein source in our high-fat/low-protein diet was also casein (22.8% [wt/vol]) (Table S2), yet that in the high-fat/high-protein diet was whey protein (50.0% [wt/vol]) (Table S1) (32). However, since casein and whey protein are both derived from milk and have similar amino acid contents (Table S1 and S3) and since they are provided as prehydrolyzed peptides, we expect that they would differ little in amino acid availability. Nevertheless, because the protein sources were not identical, further work is needed to disentangle any effects of protein source, digestibility, or abundance on CDI.

10.1128/mSystems.00765-19.8 TABLE S2 Composition of the high-fat/high-protein diet. Download Table S2, EPS file, 2 MB. Copyright © 2020 Mefferd et al. 2020 Mefferd et al. This content is distributed under the terms of the Creative Commons Attribution 4.0 International license.

10.1128/mSystems.00765-19.9 TABLE S3 Composition of the high-fat/low-protein diet. Download Table S3, EPS file, 2.1 MB. Copyright © 2020 Mefferd et al. 2020 Mefferd et al. This content is distributed under the terms of the Creative Commons Attribution 4.0 International license.

Effects of protein on CDI could be direct and/or indirect. C. difficile can use many different amino acids as Stickland donors, and both proline and glycine can be used as Stickland acceptors (9). And yet the C. difficile proline reductase (PrdA) was expressed only in humanized mice inoculated with dysbiotic microbiomes (11), suggesting that C. difficile may not compete well for proteins or amino acids with healthy microflora. In “healthy” humanized mice, PrdA was expressed by three members of the *Lachnospiraceae*, suggesting they might be important competitors for proline and other amino acids. *Lachnospiraceae* was also one of only three bacterial families that were significantly depleted in the dysbiotic humanized mice, along with *Ruminococcaceae* and *Bacteroidaceae*. In our study, most of the bacteria that contributed to microbiome variation belonged to *Lachnospiraceae* or *Ruminococcaceae*, and all of these organisms decreased in abundance in response to the diets and/or antibiotic treatment, suggesting they may be important competitors of C. difficile for amino acids in the lumen (33). Those two families are also dominant producers of butyrate in the gut (30), which directly inhibits C. difficile growth *in vitro* (10). In addition, butyrate also provides protection against CDI by improving intestinal barrier function and reducing intestinal inflammation through overexpression of hypoxia-inducible factor 1 (HIF-1) (34). *Lachnospiraceae* in particular is a dominant bacterial family in the gut microbial communities of many mammals, and many members of the family provide benefits to the host (35). A member of this family has also been documented to provide colonization resistance to C. difficile in a CDI mouse model (36) and has been shown to produce iso (3β-hydroxy) bile acids (37), which are inhibitors of C. difficile growth. Thus, it appears that members of *Lachnospiraceae* and *Ruminococcaceae* act against C. difficile through independent mechanisms, including competition for resources and production of inhibitory SCFAs and secondary bile acids. In our study, the high-fat/high-protein diet would lead to an abundance of oligopeptides and free amino acids in the lumen and could provide a selective advantage that leads to C. difficile overgrowth when coupled with the loss of *Lachnospiraceae* and *Ruminococcaceae.* Diet-induced or antibiotic-induced loss of *Lachnospiraceae* and *Ruminococcaceae* might not be a significant problem in mice fed other diets, particularly the high-carbohydrate diet, because of the low concentrations of peptides and amino acids and/or the healthful effects of SCFAs resulting from carbohydrate fermentation by other microorganisms (see above).

High-protein diets have more-general consequences for gut health that may exacerbate pathogenesis. Proteins that are not easily digested cause a surplus of dietary protein reaching the distal gut that can be acted upon by protein-fermenting bacteria. Fermentation of sulfur-containing amino acids can lead to excessive hydrogen sulfide, via desulfurlyation of cysteine and methionine, which inhibits host cytochrome *c* oxidase, damages colonic cell genomic DNA, alters cellular pathways, inhibits host-cell butyrate oxidation, and reduces the life span of colonocytes (38, 39). Production of ammonia due to protein fermentation (ammonification) can result in reduced gut health through decreased SCFA production, increased paracellular permeability of gut epithelial cells, and altered epithelial cell morphology (40, 41). And yet, the effect of the high-fat/high-protein diet alone on the health of mice does not explain the health problems in those mice, as the experimental diets were given for 10 days prior administration of antibiotics and C. difficile challenge and there were no observable changes in behavior or host health.

### Effect of a high-fat/low-protein keto-like diet on antibiotic-induced CDI.

Mice fed the high-fat/low-protein diet showed variability in disease severity and survival (Fig. 2) and highly variable microbiome compositions after antibiotic treatments (Fig. 4). Thus, the effect of fats on CDI is uncertain. The high-fat/low-protein diet was high in saturated fats, as the fat source was hydrogenated coconut oil (33.3% [wt/vol]) (Table S2). Coconut oil is >90% saturated fatty acids and consists of medium-length chains rich in lauric acid (C8:0) (42). This diet closely mimics the MCT diet, which is a version of ketogenic diet (19). Diets high in saturated fats can increase the incidence of colitis in immunocompromised mice due to increased inflammatory response (43). Such an effect points to plausible hypotheses with respect to the mechanisms by which healthy mice fed the high-fat/low-protein diet may have experienced increased CDI severity and reduced survival following C. difficile challenge. However, the high-fat diets used in these studies also had low carbohydrate content, which itself may have been an important factor in disease progression in some animals.

### Effect of diet on C. difficile pathogenesis.

In addition to the direct effects of diet on C. difficile germination, growth, and sporulation and the indirect effects mediated by ecological interactions, diet may also affect C. difficile by directly altering the expression of pathogenesis factors. Interestingly, *in vitro* studies on C. difficile strain VPI 10463 showed that a blend of nine amino acids, including proline and cysteine, downregulated toxin production (44, 45). This result superficially contradicts our results and those reported by others (11) showing that dietary proteins exacerbate CDI in mice, underscoring the complexity of CDI and the need for additional studies to better understand the ecological and molecular mechanisms governing diet-specific CDI outcomes.

### Other microbial community responses to diets, antibiotics, and CDI.

Following changes in diet, mice developed distinct microbial communities, which were then disturbed by antibiotic treatments and CDI (Fig. 4; see also Fig. S2 to S5). The shift in community composition after diet and antibiotics administration was accompanied by reduced alpha diversity associated with the effects of diet, antibiotic treatment, and CDI (Fig. 3; see also Fig. S1), which is consistent with previous work. Perturbations to gut microbial communities resulting from administration of antibiotics can result in CDI due to loss of colonization resistance, especially following a course of antibiotics, as seen in humans (46) and across CDI mouse and hamster animal models (5, 47, 48). Typically, reduced colonization resistance and increased susceptibility to CDI are marked by a decrease in gut microbial diversity (5, 49), and yet a recent report suggested that CDI susceptibility and severity are independent of alpha diversity (10). Likewise, our work showed loss of diversity in infected mice across all experimental diets (Fig. 3) and yet also showed significantly different survival and severity outcomes between the groups (Fig. 2). This demonstrates that diet plays a critical and dominant role in CDI disease patterns over microbial diversity *per se*.

In addition to the changes in *Lachnospiraceae* and *Ruminococcaceae* abundances described above, the abundances of several other microorganisms changed throughout the experiment. There was a loss of a member of the genus *Parasutterella* after a change in diet (Fig. 6). *Parasutterella* species are a part of the core gut microbiome in humans and mice, with occasional associations with disease. A study characterizing *Parasutterella* isolated from mouse guts showed that the presence of this organism significantly changed patterns of aromatic amino acid metabolism in the gut and that the organism is crucial for bile acid metabolism and homeostasis (50). This is especially important since conjugated primary bile salts are required for spore germination whereas secondary bile acids are known inhibitors of sporulation and growth of C. difficile. Therefore, *Parasutterella* could play an important role in colonization resistance through the modulation of the composition of the bile acids pool (51).

*Muribaculaceae* (formerly family S24-7) abundance decreased after administration of the high-carbohydrate and high-fat/low-protein diets (Fig. 6). Members of *Muribaculaceae* are well adapted to the mouse gut microbiome (52) and may comprise up to 685 species-level clusters (53). Further, analysis of the 157 draft genomes of the members of *Muribaculaceae* showed them to be highly enriched in glycoside hydrolases, suggesting an ability to deconstruct complex carbohydrates such as MACs and thereby promote SCFA production (53, 54).

The increase in *Alistipes* abundance after the diet changes and the reduction in abundance after antibiotic treatment might be indicative of general antibiotic susceptibility of this organism. Members of *Alistipes* are suggested to be protective against CDI (48), and depletion of these microbes has been associated with CDI (55). Another study on fecal microbiota transplant (FMT) treatments for CDI documented an increased abundance of *Alistipes* after the FMT (56). Hence, the protective effect provided by members of the genus *Alistipes* needs to be evaluated carefully.

The increased abundance of Clostridium innocuum after antibiotic treatment in all diet groups can be attributed to its ability to produce peptidoglycan precursors with a C-terminal serine, which have a low affinity for vancomycin (57). This organism’s resistance to vancomycin is a potential cause of antibiotic-associated diarrhea in humans, with effects similar to those seen with CDI (58).

Increased *Akkermansia* abundance post-C. difficile infection could be linked with increased mucus levels. In human CDI, patients with active CDI tend to secrete acidic mucins composed of MUC1 with altered oligosaccharide composition (59). We speculate that the increased abundance of *Akkermansia* postinfection (Fig. 6) could represent a result of this mucus secretion (60).

The transient surge in *Proteobacteria* abundance after antibiotic treatment in all mouse groups is a known response to loss of the dominant gut taxa (61). *Proteobacteria* induce inflammation (61) and provide an environment conducive for invasion by pathogens such as C. difficile (62). However, this increase in the abundance of members of *Proteobacteria* was also seen in mice fed the high-carbohydrate diet, indicating that blooms of *Proteobacteria* are insufficient to induce CDI. *Parabacteroides* abundance also increased in patients with recurrent CDIs (63). *Parabacteroides* produces succinate (64), which has been found to promote C. difficile growth following antibiotic treatments (65). Thus, the survival of *Parabacteroides* after antibiotic treatments could favor the proliferation of C. difficile in infected mice.

### Conclusion.

This study revealed large differences in the outcomes of CDI in an antibiotic-induced mouse model using hypervirulent strain R20291 (RT027) and that the differences were due to diets representing extremes of macronutrient composition. The poor outcome seen with mice fed an Atkins-like diet demands a close look at whether Atkins, ketogenic, or other high-fat/high-protein diets create high risk for CDI in humans, particularly if members of the *Lachnospiraceae* and *Ruminococcaceae* are disrupted by antibiotics. In contrast, the protective effect of a high-carbohydrate diet, despite high monosaccharide and digestible starch content, is inconsistent with recent reports on the possible importance of the effects of simple sugars on CDI. Since monosaccharides and digestible starch would not be expected to improve gut health, the apparent protective effect may be due to the low protein and/or fat content rather than to protective effects of carbohydrates *per se*.

## MATERIALS AND METHODS

### Bacterial growth conditions and spore harvest.

C. difficile R20291 (RT027), a gift from Nigel Minton, University of Nottingham, was grown on Bacto brain heart infusion (BHI) agar plates in an anaerobic chamber (10% CO_2_, 10% H_2_, 80% N_2_) at 37°C for 7 days. Bacterial cells and spores were collected from plates with confluent growth by flooding the plates with ice-cold, autoclaved deionized (DI) water. Spores were pelleted by centrifugation and resuspended in fresh deionized water for three wash steps. The spores were harvested by density gradient centrifugation via the use of a 20% to 50% HistoDenz gradient. The spore pellet was washed five times with autoclaved deionized water and stored at 4°C. Schaeffer-Fulton-staining was performed to determine the purity of spore harvest.

### Animals.

The animal protocol used in this study (1039564-2) was approved by the Institutional Animal Care and Use Committee (IACUC) at the University of Nevada, Las Vegas. All experiments and procedures were conducted in line with the National Institutes of Health’s guidelines in the “Guide for Care and Use of Laboratory Animals.” Bedding, water, and mouse feed were autoclaved prior to use. Weaned female C57BL/6 mice were purchased from Charles River (Wilmington, MA) and given 1 week to acclimate in the animal facility. The following protocols represent animals 5 to 8 weeks old.

### Diet specifications.

The mouse chow used for each experimental diet (see Table S1 to S4 in the supplemental material) was purchased from TestDiet and irradiated prior to shipping. The mouse chow was stored at 4°C before use. Additional diet specifications can be found in Tables S1 to S3.

### Treatment groups and experimental timeline.

Mice were caged in groups of five, and each mouse was marked with one of five colors. Feces samples were collected from each individual mouse by stroking the back of the mouse to induce defecation. Fecal samples were collected beginning at day 0 and for the duration of the 47-day experiment. All animals were fed a standard laboratory diet until day 3. Animals were randomly separated into 5 groups (Fig. 1; see also Table S1). Two groups received a standard laboratory diet (standard laboratory diet and untreated-control standard [−CDI]). Three groups received a high-carbohydrate diet, a high-fat/low-protein diet, or a high-fat/high-protein diet. All groups were fed their respective diets, and autoclaved water was available *ad libitum*. On day 13 and for the next 2 days, all groups except the standard diet (−CDI) group were given an antibiotic cocktail (*ad libitum* in sterile drinking water, changed daily) containing kanamycin (0.4 mg/ml), gentamicin (0.035 mg/ml), colistin (850 U/ml), metronidazole (0.215 mg/ml), and vancomycin (0.045 mg/ml). After antibiotic treatment, all animals were given regular drinking water for the remainder of the experiment. On day 16, all groups except the standard diet (−CDI) group were administered a single dose of intraperitoneal clindamycin (10 mg/kg of body weight) dissolved in autoclaved DI water. Animals in the standard diet (−CDI) group were administered intraperitoneal DI water, the vehicle control for clindamycin. On day 17, animals in all groups, except those in the standard laboratory diet (−CDI) group, were challenged with 10^8^ CFU of C. difficile R20291 spores by oral gavage. For 30 days following challenge, animals were monitored daily for signs of CDI. CDI severity scores were assigned following the rubric described in the paragraph below. Mice were weighed twice daily after challenge inside a biosafety hood. Animals were euthanized 30 days postinfection, or as soon as CDI symptoms reached a clinical endpoint as described below, so that no animals experienced unrelieved pain or distress.

### Scoring CDI severity.

For mice, disease signs were scored using the following rubric (amended from previously published protocols [21]): pink anogenital area, score of 1; red anogenital area, score of 2; lethargy, score of 2; diarrhea/increase in soiled bedding, score of 1; wet tail, score of 2; hunchback posture, score of 2; 8% to 15% loss of body weight, score of 1; >15% loss of body weight, score of 2. Animals scoring 2 or less were indistinguishable from noninfected controls and were considered nondiseased. Animals scoring 3 to 4, with signs consisting of pink anogenital area, lethargy, an increase of soiled bedding and minor weight loss, were considered to have mild CDI. Animals scoring 5 to 6, with signs consisting of mild CDI plus red anogenital area and hunchback posture, were considered to have moderate CDI. Animals scoring >6 were considered to have severe CDI and were immediately euthanized. Dietary changes were not expected to affect the animal negatively. However, if any signs of distress or disease were observed, procedures were carried out as indicated in the rubric. A two-way repeated-measures ANOVA was performed to determine the effect of different diets on disease severity over time. Euthanized mice were also included in the ANOVA calculation and were given a score of 7. Statistical significance was defined as represented by *P* values of <0.05.

### Illumina sequencing.

Fecal samples were collected for microbiome analysis and archived at –80°C. DNA was extracted from thawed fecal samples by the use of a QIAamp DNA stool minikit and quantified using a NanoDrop 1000 spectrophotometer. The V4 region of the 16S rRNA gene was PCR amplified using modified primers 515F (GTGYCAGCMGCCGCGGTAA) and 806R (GGACTACNVGGGTWTCTAAT), with the forward primer containing a 12-bp barcode (66). The PCR products were cleaned, quantified, and pooled at equimolar concentrations. Paired-end sequencing (151 bp by 12 bp by 151 bp) of pooled amplicons was performed using a MiSeq platform and customized sequencing primers at Argonne National Laboratory. Over 6.4 million good-quality reads of the V4 region of the 16S rRNA gene from 237 fecal samples were collected throughout the experimental timeline.

### Sequence analysis.

All sequence-based analysis was performed in QIIME 2 (version 2018.6) (67). Raw Illumina reads were demultiplexed using sample-specific barcodes; approximately 29,146 reads per sample were obtained. The sequences were denoised using the dada2-denoise-paired plugin to remove low-quality, chimeric, and artifactual sequences, and the resulting high-quality sequences were clustered into 2,657 sequence variants (SVs). Taxonomy was assigned to each SV by the use of a sklearn-based taxonomy classifier that uses a naive Bayes machine learning for classification and a classifier that was trained on the V4 region of the 16S rRNA gene “Silva 132 99% OTUs full-length sequences” database. SVs assigned to mitochondria, chloroplast, eukarya, and other unknown domains were excluded from further analysis. A phylogenetic tree of SVs was created by performing multiple-sequence alignment by the use of MAFFT software followed by constructing an unrooted tree by the use of FastTree software, and the unrooted tree was then rooted using the midpoint rooting method. The resultant tree was utilized for subsequent phylogeny-based analysis. Due to the variations in sequencing depth (2,505 to 76,203 quality-filtered sequences per sample), we performed alpha and beta diversity analyses at various rarefied sequence depths—5,000, 7,000, 10,000, and 15,000. We noted no significant differences at each of these rarified depths (data not shown). To maintain a trade-off between sampling depth and the number of samples, the data shown here were not normalized for sequencing depth. We also note that a population of C. difficile of very low abundance was observed in the microbiome of the uninfected control group on day 17, possibly representing airborne spores (see Fig. S6 in the supplemental material); however, these animals did not display any CDI signs and this population was quickly cleared.

### Analysis of the effects of diet on the gut microbiome.

Alpha diversity was estimated by calculating Shannon, Simpson, and observed SV diversity indices. ANOVA was performed to test if there were significant differences in alpha diversities in response to diet, antibiotic administration, and CDI. Further, a Tukey’s honestly significance difference test was performed on the ANOVA output to conduct pairwise comparisons of alpha diversity indices and to correct for multiple comparisons. Those data included data that were missing due to mouse death or to an inability to collect fecal samples as a consequence of disease severity. The SV table, phylogenetic tree, and associated metadata file were then imported in R (3.5.0) (68) and further analyzed using phyloseq (version 1.25.2) (69) and vegan (version 2.5.2) and were suitably visualized using ggplot2 (version 3.0.0) and Inkscape. NMDS was performed using Bray-Curtis dissimilarity, and the relationships of the samples with respect to diet, antibiotic treatment, and CDI over the course of experiment were displayed using the first two dimensions. ANOSIM was calculated using a Bray-Curtis dissimilarity matrix. SIMPER (22) was used to identify the SVs that were contributing to 50% of the observed differences in microbial communities at any time point during the experiment.

### Data availability.

Files containing the original unfiltered sequences are available from the NCBI under BioProject number PRJNA528113. All sequence data and scripts are available from GitHub at https://github.com/hedlundb/C.diff_Diet_Study.

10.1128/mSystems.00765-19.10 TABLE S4 Composition of the high-carbohydrate diet. Download Table S4, EPS file, 2.1 MB. Copyright © 2020 Mefferd et al. 2020 Mefferd et al. This content is distributed under the terms of the Creative Commons Attribution 4.0 International license.
