# Supplementary material for: A High-Fat/High-Protein, Atkins-Type Diet Exacerbates Clostridioides (Clostridium) difficile Infection in Mice, whereas a High-Carbohydrate Diet Protects
Source: mSystems. 2020 Feb 11;5(1):e00765-19. doi: 10.1128/mSystems.00765-19 (PMC7018531; doi:10.1128/mSystems.00765-19)
Supplement: TABLE S1 [file mSystems.00765-19-st001.pdf]

Table S1: Experimental setup for testing effect of diet on CDI mouse model.

Diet	Purpose	Antibiotic Cocktail	Clindamycin	*C. difficile*	% Kcal Fat	% Kcal Protein	% Kcal Carbohydrate
Standard lab diet +CDI[1]	- Control	+	+	+	13.4	29.8	56.7
Standard (-CDI)[1]	+ Control	-	-	-	13.4	29.8	56.7
High-fat/high-protein diet[2]	Experimental	+	+	+	54	40	6
Low-fat/high-carbohydrate diet[3]	Experimental	+	+	+	11.9	15.9	72.2
High-fat/low-protein diet[4]	Experimental	+	+	+	59.3	14.9	25.8

References/Description

1: LabDiet 5001

2: Custom purified diet containing approximately 50% protein and 25% fat (TestDiet Catalog # 1818123-286; Freudenberg et al. 2012)

3: Modified from AIN-93G Low Fat Control Diet for the Western Diet- TestDiet 5TJN (TestDiet Catalog # 1818122-203)

4: AIN-76A Semi-Purified Diet 5800-B Surwit Diet with 58% Fat Energy/Sucrose (TestDiet Catalog # 1818121-286)
